# Small molecules restore the function of mutant CLC5 associated with Dent disease

**DOI:** 10.1111/jcmm.16091

**Published:** 2020-11-16

**Authors:** Jingshu Liu, Tal T. Sadeh, Jonathan D. Lippiat, Rajesh V. Thakker, Graeme C. Black, Forbes Manson

**Affiliations:** ^1^ Division of Evolution and Genomic Sciences Faculty of Biology, Medicine and Health University of Manchester Manchester UK; ^2^ School of Biomedical Sciences Faculty of Biological Sciences University of Leeds Leeds UK; ^3^ Academic Endocrine Unit Oxford Centre for Diabetes Endocrinology &Metabolism (OCDEM) Churchill Hospital University of Oxford Oxford UK; ^4^ Manchester Centre for Genomic Medicine Manchester Academic Health Sciences Centre Manchester University NHS Foundation Trust St Mary’s Hospital Manchester UK

**Keywords:** 2‐naphthoxyacetic acid (2‐NOAA), 4‐phenylbutyrate (4PBA), CLC5, *CLCN5*, dent disease

## Abstract

Dent disease type 1 is caused by mutations in the *CLCN5* gene that encodes CLC5, a 2Cl^−^/H^+^ exchanger. The CLC5 mutants that have been functionally analysed constitute three major classes based on protein expression, cellular localization and channel function. We tested two small molecules, 4‐phenylbutyrate (4PBA) and its analogue 2‐naphthoxyacetic acid (2‐NOAA), for their effect on mutant CLC5 function and expression by whole‐cell patch‐clamp and Western blot, respectively. The expression and function of non‐Class I CLC5 mutants that have reduced function could be restored by either treatment. Cell viability was reduced in cells treated with 2‐NOAA. 4PBA is a FDA‐approved drug for the treatment of urea cycle disorders and offers a potential therapy for Dent disease.

## 1 INTRODUCTION

Dent disease is an X‐linked recessive kidney disease characterized by low‐molecular‐weight proteinuria (LMWP), hypercalciuria, aminoaciduria, nephrolithiasis and nephrocalcinosis.^1^ Two types are recognized: Dent disease type 1 is caused by *CLCN5* mutations (50%‐60% incidence) and type 2 is caused by *OCRL* mutations (15% incidence).^2^, ^3^ The *CLCN5* gene encodes a homodimeric 2Cl^−^/H^+^ exchanger, CLC5, which is mainly expressed on early endosomal membranes of cells of renal proximal tubules and the thick ascending limb of Henle's loop, suggesting a role in the receptor‐mediated endocytic pathway responsible for protein absorption.^4^

More than 200 *CLCN5* mutations are reported in Dent disease patients, and ~25% have been functionally analysed and classified into three classes according to their impact at the cellular and molecular levels.^5^ Class I mutations cause protein misfolding, being retained in the endoplasmic reticulum (ER) and degraded by the proteasome. Class II mutations result in delayed protein processing and reduced protein stability with decreased cell surface expression and function, but with normal distribution in early endosomes. Class III mutations have no influence on protein localization in early endosomes or at the cell surface but do cause reduced or abolished Cl^−^ conductance. A minority of mutations do not fit into any of these three classes, reflecting the limitation of the classification and the need for further functional analysis.

No drugs targeting the molecular defects of Dent disease have been identified. Small molecules such as 4‐phenylbutyrate (4PBA) are able to functionally restore a range of channelopathies associated with protein misfolding/instability (eg cystic fibrosis, bestrophinopathies).^6^, ^7^ We tested the effect of 4PBA and its analogue 2‐naphthoxyacetic acid (2‐NOAA) on the expression and function of mutant CLC5.

## 2 MATERIALS AND METHODS

Detailed materials and methods are provided in the online Supporting Information section.

## 3 RESULTS

### 3.1 Effect of 4PBA and 2‐NOAA on CLC5 function

Wild type and 6 previously reported disease‐causing mutant CLC5 proteins were tested.^8^ Among them, p.S270R, p.G513E, p.R516W and p.I524K are Class I mutations that cause ER retention of the mutant proteins and absence on the early endosome or plasma membranes, resulting in abolished Cl^−^ currents by patch‐clamp analysis. The other two mutants, p.G57V and p.R280P, have reduced, but not abolished, function and plasma membrane expression compared to wild‐type protein. The p.R280P mutant also expressed in early endosomes that assign it to Class II mutation, while the p.G57V mutant was found in late endosomes, making it unclassified to any of the mutation classes described above.^5^, ^8^

**Figure 1:**
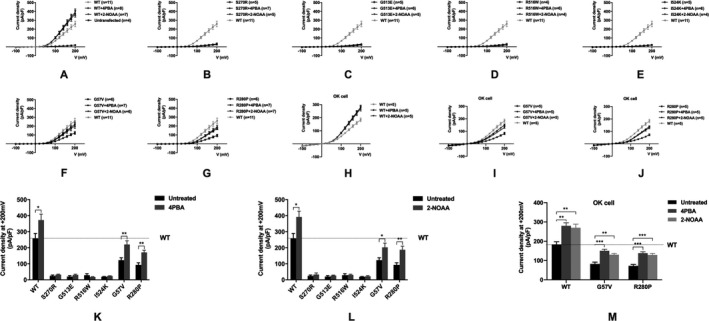
Effect of 4PBA and 2‐NOAA on CLC5 function. A–G, I‐V relationships of wild type and each mutant CLC5 with or without 4PBA or 2‐NOAA treatment in HEK293T cells. H‐J, I‐V relationships of wild‐type and non‐Class I mutant CLC5 with or without 4PBA or 2‐NOAA treatment in OK cells. K, Current densities at +200 mV of wild type and mutant CLC5 with 4PBA treatment in HEK293T cells. L, Current densities at +200 mV of wild type and mutant CLC5 with 2‐NOAA treatment in HEK293T cells. M, Current densities at +200 mV of wild type and non‐Class I mutant CLC5 with 4PBA or 2‐NOAA treatment in OK cells. Data are presented as mean ± s.e.m. *P < .05, **P < .01 and ***P < .001 indicate significant differences between groups. WT = wild type. n = number of cells recorded

Transfected HEK293T cells expressing wild type or mutant CLC5 were treated with 2.5 mmol/L 4PBA or 2‐NOAA for 24 hours before patch‐clamp analysis. Wild‐type CLC5 gave robust Cl^−^ currents with a strong outwardly rectifying current‐voltage (I‐V) relationship that was activated above +30 mV. All Class I mutants showed abolished Cl^−^ conductance that was indistinguishable from untransfected HEK293T cells. p.G57V and p.R280P had a reduced Cl^−^ conductance compared to wild‐type protein. 4PBA and 2‐NOAA had no effect on any of the Class I mutants but both significantly increased the Cl^−^ conductance of wild type (44% increase with 4PBA and 52% increase with 2‐NOAA) and non‐Class I mutants (p.G57V: +4PBA 48% to 85% of wild type, +2‐NOAA 48% to 78%; p.R280P: +4PBA 36% to 66% of wild type, +2‐NOAA 36% to 72%) (Figure 1A–G, K and L). These findings were then validated in opossum kidney (OK) cells derived from proximal tubule epithelium, the endogenous cell that CLC5 is normally expressed in (Figure 1H–J, M). These data show that the small molecules 4PBA and 2‐NOAA can restore Cl^−^ conductance to non‐Class I CLC5 mutants that can exit the ER.

### 3.2 Effect of 4PBA and 2‐NOAA on CLC5 expression

**Figure 2:**
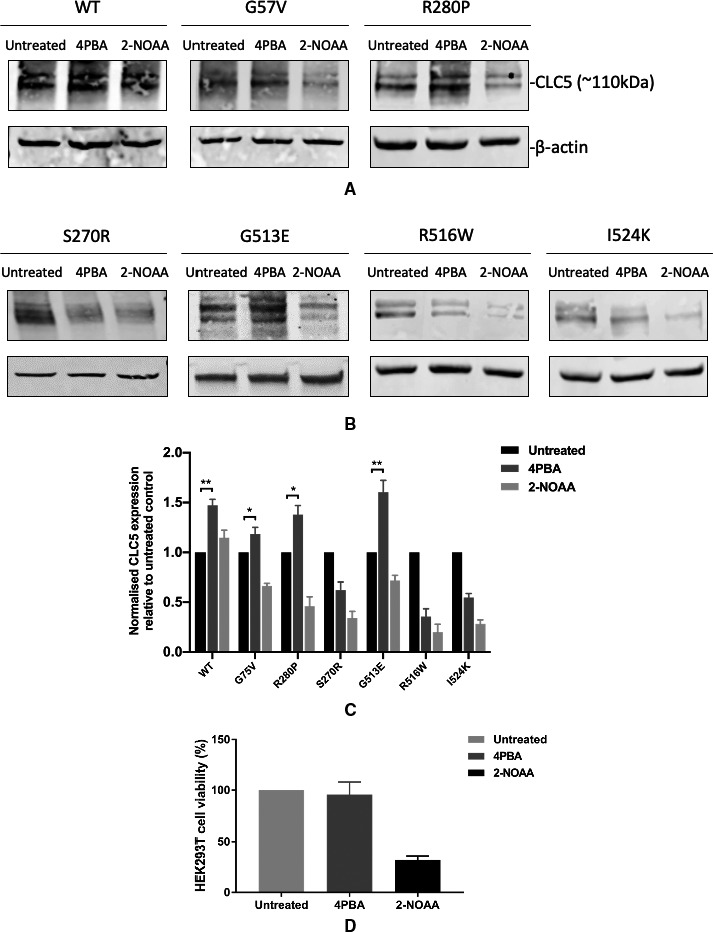
Effect of 4PBA and 2‐NOAA on CLC5 expression and HEK293T cell viability. A, Representative Western blots of wild type and non‐Class I mutant CLC5 (p.G57V and p.R280P) with 4PBA or 2‐NOAA treatment. B, Representative Western blots of Class I mutant CLC5 (p.S270R, p.G513E, p.R516W and p.I524K) with 4PBA or 2‐NOAA treatment. C, Quantification of Western blot data from 3 independent experiments is presented as mean ± s.e.m. *P < .05 and **P < .01 indicate significant differences between groups. The untreated controls are taken as 1. D, HEK293T cell viability with or without treatment (2.5 mmol/L 4PBA or 2‐NOAA treatment for 24 hours) determined by MTT assay. Untreated group is taken as 100% in the graph. Data were collected from 3 independent experiments and are presented as mean ± s.e.m. WT = wild type

In order to investigate whether the increased function is due to increased CLC5 expression, transfected HEK293T cells were treated with 2.5 mmol/L 4PBA or 2‐NOAA for 24 hours before harvesting for Western blotting. Anti‐GFP antibody was used to detect YFP‐tagged CLC5 and anti‐β‐actin antibody was used as a loading control. The full‐length, glycosylated CLC5‐YFP protein (110 kDa) was detected in lysates from cells expressing wild type or mutant CLC5. 4PBA treatment significantly increased the expression of wild type, p.G57V, p.R280P and p.G513E CLC5 proteins. 2‐NOAA did not increase the expression of either the wild type or mutant proteins, which may be partially explained by the notable cell death during the 24 hours treatment period (Figure 2A–C).

### 3.3 Effect of 4PBA and 2‐NOAA on HEK293T cell viability

Cell death was observed in cell cultures treated with 2‐NOAA, and the reduced expression of some proteins seen by Western blotting led to the suspicion that 2‐NOAA was cytotoxic to HEK293T cells. The MTT cell viability assay was used to determine the cytotoxicity of 2.5 mmol/L 4PBA and 2‐NOAA on HEK293T cells. Results are shown as cell viability (%) relative to control (untreated) group. 4PBA had no significant effect on cell viability. 2‐NOAA treatment caused the death of more than half the cells, with a cell viability of 32% compared to the untreated cells (Figure 2D).

## 4 DISCUSSION

CLC5 is a 2Cl^−^/H^+^ exchanger mainly expressed on the early endosome membrane and facilitates the endocytosis of low‐molecular‐weight proteins in proximal tubules, corresponding with the key clinical feature of Dent disease, LMWP. Similar to other protein misfolding diseases, the majority of CLC5 mutants studied to date cause decreased expression of the mature protein, rapid degradation by the proteasome, mislocalization and reduced or abolished Cl^−^ conductance.^5^ 4PBA is an FDA‐approved drug used for the treatment of urea cycle disorders, where it provides an alternative pathway to the urea cycle for the excretion of excess nitrogen. It can also act as a chemical chaperone that assists protein folding, and as a histone deacetylase inhibitor that regulates gene transcription (eg up‐regulates stress response gene expression).^9^ 2‐NOAA, a 4PBA analogue, has been shown to be more potent in suppressing ER stress compared to 4PBA.^10^ The cell electrophysiology showed that only the function of the p.G57V and p.R280P mutants that exit the ER were rescued by the addition of 4PBA or 2‐NOAA. None of the Class I mutants that are retained in the ER were functionally rescued. These effects were seen in both HEK293T cells and the proximal tubule epithelium endogenous cell type. These data were supported by the Western blot experiment that showed the expression of p.G57V and p.R280P was increased by small molecule treatment. As the ER‐retained Class I mutant proteins may have more serious defects in protein folding compared to non‐Class I CLC5 mutants that are released from the ER, the former would be expected to be more difficult, or impossible, to functional rescue by small molecules. A similar finding was noted in the ivacaftor treatment of mutant CFTR associated with cystic fibrosis where the amount of therapeutic effect correlated with the degree of disruption in CFTR protein processing and/or channel function.^11^

In conclusion, our data demonstrate that non‐Class I CLC5 protein mutated in Dent disease type 1 can be functionally rescued by small molecule treatment. The absence of any functional rescue of Cl^−^ conductance to Class I mutants suggests that different pathogenic mechanisms result from different classes of mutation and that these are important considerations when contemplating small molecule treatment. 2‐NOAA treatment was associated with substantial toxicity, indicating that therapeutic safety can differ dramatically between compounds with minimal structural differences. Future work will investigate the effect of 4PBA on the physiological function of CLC5 such as endosomal acidification and endocytosis.

## CONFLICT OF INTEREST

The authors confirm that there are no conflicts of interest.

## AUTHOR CONTRIBUTIONS

**Jingshu Liu:** Data curation (equal); Formal analysis (lead); Investigation (equal); Methodology (equal); Validation (equal); Visualization (lead); Writing‐original draft (lead). **Tal Thomas Sadeh:** Data curation (equal); Formal analysis (equal); Investigation (equal); Methodology (equal); Validation (equal); Visualization (supporting); Writing‐review & editing (supporting). **Jonathan D Lippiat:** Conceptualization (equal); Methodology (lead); Resources (equal); Writing‐review & editing (equal). **Rajesh V Thakker:** Conceptualization (equal); Resources (supporting); Writing‐review & editing (equal). **Graeme C Black:** Conceptualization (equal); Funding acquisition (lead); Project administration (lead); Resources (supporting); Supervision (lead); Writing‐review & editing (supporting). **Forbes Manson:** Conceptualization (equal); Project administration (lead); Resources (lead); Supervision (lead); Writing‐review & editing (lead).

## Supporting information

Supplementary material

## Data Availability

The data that support the findings of this study are available from the corresponding author upon reasonable request.
