# Supplementary material for: Small molecules restore the function of mutant CLC5 associated with Dent disease
Source: J Cell Mol Med. 2020 Nov 16;25(2):1319–22. doi: 10.1111/jcmm.16091 (PMC7812281; doi:10.1111/jcmm.16091)
Supplement: Supplementary file 1 — Supplementary material [file JCMM-25-1319-s001.docx]

**Supporting Information**

**Materials and Methods**

**Cell culture**

HEK293T cells were cultured in Dulbecco’s modified Eagle’s medium containing 10% (v/v) heat-inactivated foetal bovine serum and 2mM L-glutamine. Opossum kidney (OK) proximal tubule epithelium cells were purchased from ATCC (ATCC-CRL-1840) and cultured in Dulbecco's modified Eagle’s medium nutrient mixture F-12, supplemented with 10% (v/v) heat-inactivated foetal bovine serum and 2mM L-glutamine. Cells were incubated at 37℃, 5% CO_2_.

**Transient transfection**

HEK293T or OK cells were transfected with wildtype or mutant CLC5-YFP constructs using Fugene HD transfection reagent (Promega, Southampton, UK). All CLC5-YFP constructs are described in Smith *et al.* (2009) ^1^. The transfection medium was replaced with culture medium, with or without treatment, after 24 h and incubated for a further 24 h before use.

**Small molecules treatment**

Compounds were purchased from Sigma-Aldrich. 2.5mM of each was added to cell culture media 24h prior to harvesting for western blot or patch-clamp analysis.

**Whole-cell patch-clamp**

Transfected HEK293T or OK cells expressing CLC5-YFP were dissociated and seeded on poly-L-lysine coated 13mm glass coverslips for 24h in cell culture medium with or without treatment before analysis. During patch-clamp recording, the coverslip was placed in a chamber filled with extracellular solution (140mM CsCl, 1mM CaCl_2_, 1mM MgCl_2_, 10mM HEPES, 10mM CsOH, pH 7.4 with HCl). Glass capillaries “GC100F-10” (Harvard Apparatus, Edenbridge, UK) were pulled with a Model P-97 pipette puller (Sutter Instrument CO., USA) and fire polished to a resistance of 2-3MΩ. The pipettes were filled with intracellular solution (42mM CsCl, 49mM Cs_2_SO_4_, 10mM EGTA, 10mM HEPES, 10mM CsOH, pH 7.4 with CsOH). Cell membrane potential was held at -30mV for HEK293T cells and -20mV for OK cells before being stepped from -100mV to +200mV in 20mV increments when recording. The step duration was 1s and at least 3 recordings were taken from each cell. Conventional whole-cell recordings were performed and analysed by pCLAMP 10 (Molecular Devices, Wokingham, UK) through a MultiClamp 700A amplifier and a Digidata 1440A digitizer (Molecular Devices, Wokingham, UK). Whole-cell patch-clamp raw data were processed by Clampfit 10.7 (Molecular Devices, Wokingham, UK).

**SDS-PAGE and western blot**

Cells were lysed in RIPA buffer and lysates were loaded onto a 4-20% Mini PROTEAN TGX Stain-Free Gel (Bio-Rad, Watford, UK). Gels were transferred onto nitrocellulose membrane (LI-COR Biosciences, Cambridge, UK) by wet transfer at 300mA for 1h. The membrane was blocked in 5% milk in TBS-T (Tris-buffer saline, 0.1% Tween 20) at room temperature (RT) for 1h and then incubated with primary antibodies in 2% milk TBS-T for 1h at RT. After three washes in TBS-T, membranes were incubated with secondary antibodies in 2% milk TBS-T for 1h at RT and then washed three times in TBS-T before imaging. Antibodies are listed in Table S1. An anti-β-actin antibody was used as loading control. The LI-COR Odyssey CLx system was used to visualize the membrane and LI-COR Image Studio 5.0 to analyse the image.

**Table S1.** Antibodies used in this study

Primary antibodies

| Target | Host species | Dilution | Supplier | Catalogue No. |
| --- | --- | --- | --- | --- |
| GFP | Mouse | 1:1000 | Life Technologies | MA515256 |
| β-actin | Rabbit | 1:2000 | Abcam | Ab8227 |

Secondary antibodies

| Antibody | Target | Host species | Dilution | Supplier | Catalogue No. |
| --- | --- | --- | --- | --- | --- |
| IRDye^®^ 680RD | Mouse IgG | Donkey | 1:5000 | LI-COR | 925-68072 |
| IRDye^®^ 800CW | Rabbit IgG | Donkey | 1:5000 | LI-COR | 926-32213 |

**MTT assay**

HEK293T cells were seeded in 24-well plates at a density of 5x10^4^ cells/well 16h before treatment (2.5mM 4PBA or 2-NOAA for 24h). 0.5mg/ml MTT (3-(4,5-Dimethylthiazol-2-yl)-2,5-diphenyltetrazolium bromide) was added to the cell culture medium followed by 2h incubation at 37℃. Dimethyl sulfoxide was then added to the cell to dissolve the purple formazan product. The absorbance at 562nm was measured using BioTek ELx800 Absorbance Microplate Reader (BioTek Instruments, USA). Triplicate wells were assayed for each group including untreated control and blank control (empty wells with no cells) that was subtracted in the calculation.

**Data analysis**

Quantitative data were collected from at least 3 independent experiments and shown in graphs as mean ± standard error of the mean (s.e.m.). Statistical analysis was performed by Student’s t-test by Graphpad Prism 7.0 (Graphpad, La Jolla, USA) and statistically significant difference was defined as p<0.05.

**Reference**

1. Smith AJ, Reed AA, Loh NY, Thakker RV, Lippiat JD. Characterization of Dent's disease

mutations of CLC-5 reveals a correlation between functional and cell biological consequences and

protein structure. Am J Physiol Renal Physiol. 2009;296:F390-397.
